# $L_{p}$-convergence, complete convergence, and weak laws of large numbers for asymptotically negatively associated random vectors with values in $\mathbb{R}^{d}$

**DOI:** 10.1186/s13660-018-1699-6

**Published:** 2018-05-08

**Authors:** Mi-Hwa Ko

**Affiliations:** Division of Mathematics and Informational Statistics, Wonkwang University, Jeonbuk, Korea

**Keywords:** 60F15, Asymptotically negative association, Random vectors, Rosenthal-type inequality, $L_{p}$-convergence, Complete convergence, Weak laws of large numbers

## Abstract

In this paper, based on the Rosenthal-type inequality for asymptotically negatively associated random vectors with values in $\mathbb{R}^{d}$, we establish results on $L_{p}$-convergence and complete convergence of the maximums of partial sums are established. We also obtain weak laws of large numbers for coordinatewise asymptotically negatively associated random vectors with values in $\mathbb{R}^{d}$.

## Introduction

Ko et al. [1] introduced the concept of negative association (NA) for $\mathbb{R}^{d}$-valued random vectors.

### Definition 1.1

(Ko et al. [1])

A finite sequence $\{X_{1},\ldots ,X_{m}\}$ of $\mathbb{R}^{d}$-valued random vectors is said to be negatively associated (NA) if for any disjoint nonempty subsets $A, B\subset \{1,\ldots ,m\}$ and any nondecreasing functions *f* on $\mathbb{R}^{\vert A \vert d}$ and *g* on $\mathbb{R}^{\vert B \vert d}$,

1.1 $$ \operatorname{Cov}\bigl(f(X_{i}, i\in A),g(X_{j},j\in B) \bigr)\leq 0 $$

whenever the covariance exists. Let $\vert A \vert $ denote the cardinality of a set *A*. An infinite sequence $\{X_{i}, i\geq 1\}$ of $\mathbb{R}^{d}$-valued random vectors is negatively associated if every finite subsequence is negatively associated.

In the case of $d=1$, the concept of negative association had already been introduced by Joag-Dev and Proschan [2]. A number of well-known multivariate distributions possess the NA property, such as the multinomial distribution, multivariate hypergeometric distribution, negatively correlated normal distribution, and joint distribution of ranks.

In addition to Definition 1.1, for random vectors in $\mathbb{R}^{d}$, we can define asymptotically negative association (ANA).

### Definition 1.2

A sequence $\{X_{1},\ldots ,X_{m}\}$ of $\mathbb{R}^{d}$-valued random vectors is said to be asymptotically negatively associated (ANA) if

1.2 $$ \rho^{-}(r)=\sup_{S,T}\bigl\{ \rho^{-}(S,T):S,T \subset {\mathrm{N}}, \operatorname{dist}(S,T) \geq r\bigr\} \rightarrow 0\quad \mbox{as }r\rightarrow \infty, $$

where $\operatorname{dist}(S,T)=\min \{\vert x-y \vert ; x\in S, y\in T\}$,

$$ \rho^{-}(S,T)=0 \vee \biggl\{ \frac{\operatorname{Cov}(f(X_{i}, i\in S),g(X_{j}, j\in T))}{(\operatorname{Var} f(X_{i}, i\in S))^{\frac{1}{2}}(\operatorname{Var} g(X_{j}, j\in T))^{ \frac{1}{2}}}\biggr\} , $$

and *f* on $\mathbb{R}^{\vert S \vert d}$ and *g* on $\mathbb{R}^{\vert T \vert d}$ are any real coordinatewise nondecreasing functions.

In the case of $d=1$, the concept of asymptotically negative association was proposed by Zhang [3, 4] and studied by Yuan and Wu [5].

It is obvious that a sequence of asymptotically negatively associated random variables is negatively associated if and only if $\rho^{-}(1)=0$. Compared to negative association, asymptotically negative association defines a strictly larger class of random variables (for detailed examples, see Zhang [3, 4]). Consequently, the study of the limit theorems for asymptotically negatively associated random variables is of much interest.

We refer to Zhang [4] for the central limit theorem, Wang and Lu [6] for some inequalities of maximums of partial sums and weak convergence, Wang and Zhang [7] for the Berry–Esseen theorem and the law of the iterated logarithm, Yuan and Wu [5] for the $L_{p}$-convergence and complete convergence of the maximums of the partial sums, among others.

The concept of coordinatewise negative association (CNA) for random vectors with values in $\mathbb{R}^{d}$ was introduced as follows. Let $\langle \cdot ,\cdot \rangle $ denote the inner product, and let $\{e_{j}, j\geq 1\}$ be an orthonormal basis. A sequence $\{X_{n}, n \geq 1\}$ of $\mathbb{R}^{d}$-valued random vectors is said to be coordinatewise negatively associated (CNA) if for each *j* $(1\leq j \leq d)$, the sequence $\{X_{n}^{(j)}, n\geq 1, 1\leq j\leq d\}$ of random variables is NA, where $X_{n}^{(j)}=\langle X_{n}, e_{j}\rangle $.

As in the definition of CNA, we can define coordinatewise asymptotically negative association for random vectors with values in $\mathbb{R} ^{d}$.

### Definition 1.3

A sequence $\{X_{n}, n\geq 1\}$ of $\mathbb{R}^{d}$-valued random vectors is said to be coordinatewise asymptotically negatively associated (CANA) if for each *j* $(1\leq j\leq d)$, the sequence $\{X_{n}^{(j)}, n\geq 1, 1\leq j \leq d\}$ of random variables is asymptotically negatively associated, where $X_{n}^{(j)} =\langle X_{n}, e_{j}\rangle $ for $n\geq 1$ and $1\leq j\leq d$.

It is clear that if a sequence of $\mathbb{R}^{d}$-valued random vectors is ANA, then it is CANA. However, in general, the converse is not true.

Let $\{X, X_{n}, n\geq 1\}$ be a sequence of $\mathbb{R}^{d}$-valued random vectors. We consider the following inequalities for $1\leq j \leq d$:

1.3 $$ C_{1} P\bigl(\bigl\vert X^{(j)} \bigr\vert >t\bigr)\leq \frac{1}{n}\sum_{k=1}^{n} P\bigl(\bigl\vert X_{k}^{(j)} \bigr\vert >t\bigr) \leq C_{2}P\bigl(\bigl\vert X^{(j)} \bigr\vert >t \bigr). $$

If there exists a positive constant $C_{1}$, $(C_{2})$ such that the left-hand (right-hand) side of (1.3) is satisfied for all $1\leq j \leq d$, $n\geq 1$, and $t\geq 0$, then the sequence $\{X_{n}, n \geq 1\}$ is said to be coordinatewise weakly lower (upper) bounded by *X*. The sequence $\{X_{n}, n\geq 1\}$ is said to be coordinatewise weakly bounded by *X* if it is both coordinatewise lower and upper bounded by *X* (see Huan et al. [8]).

In Sect. 2, we give some lemmas, which will be used to prove the main results, and in Sect. 3, we prove the $L_{p}$-convergence and complete convergence results for the maximums of the partial sums of the sequence of ANA random vectors with values in $\mathbb{R}^{d}$. In addition, in Sect. 4, we establish a weak law of large numbers for CANA random vectors with values in $\mathbb{R}^{d}$.

Throughout the paper, the symbol *C* denotes a generic constant $(0< C<\infty )$, which is not necessarily the same in each occurrence, $S_{n}=\sum_{i=1}^{n} X_{i}$ for a sequence $\{X_{n}, n\geq 1\}$ of random vectors, and $\Vert \cdot \Vert _{p}$ denotes the $L_{p}$-norm. Moreover, ≪ represents the Vinogradov symbol *O*, and $I(\cdot )$ is the indicator function.

## Some lemmas

From the definition of a sequence of ANA random vectors, we have the following:

### Lemma 2.1

(Yuan and Wu [5])

*Nondecreasing* (*or nonincreasing*) *functions defined on disjoint subsets of a sequence* $\{X_{n}, n\geq 1\}$ *of ANA random vectors with mixing coefficients* $\rho^{-}(s)$ *is also ANA with mixing coefficients not greater than* $\rho^{-}(s)$.

Wang and Lu [6] proved the following Rosenthal-type inequality for a sequence of ANA random variables in $\mathbb{R}^{1}$.

### Lemma 2.2

*For a positive integer* $N\geq 1$, *positive real numbers* $p\geq 2$, *and* $0\leq r <(\frac{1}{6p})^{p/2}$, *if* $\{X_{i}, i \geq 1\}$ *is a sequence of ANA random variables with* $\rho^{-}(N) \leq r$, $EX_{i}=0$, *and* $E\vert X_{i} \vert ^{p}<\infty $ *for every* $i\geq 1$, *then there is a positive constant* $D=D(p,N,r)$ *such that*, *for all* $n\geq 1$,

2.1 $$ E\max_{1\leq k\leq n}\Biggl\vert \sum_{i=1}^{k} X_{i} \Biggr\vert ^{p} \leq D\Biggl(\sum _{i=1}^{n} E\vert X_{i} \vert ^{p}+\Biggl(\sum_{i=1}^{n} E \vert X_{i} \vert ^{2}\Biggr)^{p/2} \Biggr). $$

Inspired by the proof of Lemma 2.3 in Li-Xin Zhang [9], we extend Lemma 2.2 to $\mathbb{R}^{d}$-valued ANA random vectors as follows.

### Lemma 2.3

*For a positive integer* $N\geq 1$, *positive real numbers* $p\geq 2$, *and* $0\leq r<(\frac{1}{6p})^{p/2}$, *if* $\{X_{i}, i \geq 1\}$ *is a sequence of* $\mathbb{R}^{d}$-*valued ANA random vectors with* $\rho^{-}(N)\leq r$, $EX_{i}=0$, *and* $E\Vert X_{i} \Vert ^{p}<\infty $ *for every* $i\geq 1$, *then there is a positive constant* $D^{\prime}=D^{\prime}(p, N, r)$ *such that*, *for all* $n\geq 1$,

2.2 $$\begin{aligned}& E\max_{1\leq k\leq n} \Biggl\Vert \sum_{i=1}^{k} X_{i} \Biggr\Vert ^{p} \\& \quad \leq D^{\prime}\Biggl(\sum _{i=1} ^{n} E\Vert X_{i} \Vert ^{p}+\Biggl(\sum_{i=1}^{n} E\Vert X_{i} \Vert ^{2}\Biggr)^{p/2} \Biggr). \end{aligned}$$

### Proof

Note that

$$ \max_{1\leq k\leq n}\Biggl\Vert \sum_{i=1}^{k} X_{i} \Biggr\Vert =\max_{1\leq k\leq n} \sum _{j=1}^{d} \Biggl\vert \sum _{i=1}^{k}X_{i}^{(j)} \Biggr\vert \leq \sum_{j=1}^{d} \max _{1\leq k\leq n} \Biggl\vert \sum_{i=1}^{k}X_{i}^{(j)} \Biggr\vert $$

and by Lemma 2.2

$$\begin{aligned} E\Biggl(\max_{1\leq k\leq n}\Biggl\vert \sum _{i=1}^{k} X_{i} \Biggr\vert ^{p}\Biggr) \leq &D\Biggl(\Biggl(\sum_{i=1} ^{n}E\bigl\vert X_{i}^{(j)} \bigr\vert ^{2}\Biggr)^{\frac{p}{2}}+\sum_{i=1}^{n} E\bigl\vert X_{i}^{(j)} \bigr\vert ^{p}\Biggr) \\ \leq &D\Biggl(\Biggl(\sum_{i=1}^{n}E \Vert X_{i} \Vert ^{2}\Biggr)^{\frac{p}{2}}+\sum _{i=1}^{n} E \Vert X_{i} \Vert ^{p}\Biggr). \end{aligned}$$

Hence (2.2) follows. □

From Lemma 1.2 of Kuczmaszewska [10] we obtain the following lemma.

### Lemma 2.4

*Let* $\{X_{n}, n\geq 1\}$ *be a sequence of* $\mathbb{R}^{d}$-*valued random vectors weakly upper bounded by a random vector* *X*, *and let* $r>0$. *Then*, *for some constant* $C>0$, $E\Vert X \Vert ^{r}< \infty $ *implies* $n^{-1}\sum_{k=1}^{n} E\Vert X_{k} \Vert ^{r}\leq CE\Vert X \Vert ^{r}$.

The following lemma supplies us with the analytical part in the proofs of the theorems in the subsequent sections.

### Lemma 2.5

(Yuan and Wu [5])

*Let* $\{a_{n}, n\geq 1\}$ *and* $\{b_{n}, n\geq 1\}$ *be sequences of nonnegative numbers*. *If*

$$ \sup_{n\geq 1} n^{-1}\sum_{i=1}^{n} a_{i}< \infty \quad \textit{and}\quad \sum_{n=1}^{\infty }b_{n}< \infty , $$

*then*

2.3 $$ \sum_{i=1}^{n} a_{i}b_{i} \leq \Biggl(\sup_{m\geq 1}m^{-1}\sum _{i=1}^{m} a _{i}\Biggr)\sum _{i=1}^{n} b_{i} $$

*for every* $n\geq 1$.

Next, we will extend some $L_{p}$-convergence and complete convergence results for the maximums of the partial sum of $\mathbb{R}^{1}$-valued ANA random variables in Yuan and Wu [5] to $\mathbb{R}^{d}$-valued random vectors.

## $L_{p}$-convergence and complete convergence for ANA random vectors with values in $\mathbb{R}^{d}$

The following theorem is an extension of Theorem 3.2 in Yuan and Wu [5] to random vectors with values in $\mathbb{R}^{d}$.

### Theorem 3.1

*Let* $p\geq 2$ *be positive real numbers*, *and let* $N\geq 1$ *be a positive integer*. *Suppose that* $\{X_{n}, n\geq 1\}$ *is a sequence of* $\mathbb{R}^{d}$-*valued ANA random vectors with mixing coefficients* $\rho^{-}(s)$ *such that* $\rho^{-}(N)<(\frac{1}{6p})^{p/2}$. *If* $\{X_{n}, n\geq 1\}$ *are* $\mathbb{R}^{d}$-*valued random vectors satisfying*

3.1 $$ \sup_{n\geq 1}\frac{1}{n}\sum_{k=1}^{n} E\Vert X_{k} \Vert ^{p}< \infty , $$

*then*, *for any* $\delta >\frac{1}{2}$,

3.2 $$ n^{-\delta }\max_{1\leq i \leq n}\Vert S_{i}-ES_{i} \Vert \rightarrow 0\quad \textit{in } L_{p}. $$

### Proof

By Lemma 2.3, the Hölder inequality, and (3.1) we obtain

3.3 $$\begin{aligned} &E\Bigl(n^{-\delta }\max_{1\leq i\leq n}\Vert S_{i}-ES_{i} \Vert \Bigr)^{p} \\ &\quad = n^{-p\delta }E\max_{1\leq i \leq n}\Biggl\Vert \sum _{j=1}^{i}(X_{j}-EX_{j}) \Biggr\Vert ^{p} \\ &\quad \ll n^{-p\delta }\sum_{i=1}^{n} E \Vert X_{i}-EX_{i} \Vert ^{p}+n^{-p\delta} \Biggl(\sum_{i=1}^{n} E\Vert X_{i}-EX_{i} \Vert ^{2}\Biggr)^{p/2} \quad\bigl(\mbox{by (2.2)}\bigr) \\ &\quad \ll n^{-p\delta -1+\frac{p}{2}}\sum_{i=1}^{n} E\Vert X_{i} \Vert ^{p} \\ &\quad \ll n^{-p\delta +\frac{p}{2}}\sup_{n\geq 1} \frac{1}{n} \sum _{i=1} ^{n}E\Vert X_{i} \Vert ^{p}, \end{aligned}$$

which by Lemma 2.5 yields (3.2) for any $\delta >\frac{1}{2}$. □

As applications of Theorem 3.1, we introduce two results that are not present in Yuan and Wu [5].

### Theorem 3.2

*Let* $p\geq 2$ *be positive real numbers*, *and let* $N\geq 1$ *be a positive integer*. *Suppose that* $\{X_{n}, n\geq 1\}$ *is a sequence of* $\mathbb{R}^{d}$-*valued ANA random vectors with mixing coefficients* $\rho^{-}(s)$ *such that* $\rho^{-}(N)<(\frac{1}{6p})^{p/2}$. *If* $\{X_{n}, n\geq 1\}$ *is weakly upper bounded by a random vector* *X* *with* $E\Vert X \Vert ^{p}<\infty $, *then*, *for any* $\delta >\frac{1}{2}$, (3.2) *holds*.

### Proof

By Lemma 2.3, Lemma 2.4(i), Hölder’s inequality, and the proof of Theorem 3.1 we obtain

$$\begin{aligned}& E\Bigl(n^{-p \delta }\max_{1\leq i\leq n}\Vert S_{i}-ES_{i} \Vert \Bigr)^{p} \\& \quad \leq n^{-p\delta -1+\frac{p}{2}}\sum_{i=1}^{n} E \Vert X_{i} \Vert ^{p} \quad \bigl(\mbox{see (3.3)}\bigr) \\& \quad \leq n^{-p\delta +\frac{p}{2}}E\Vert X \Vert ^{p}\rightarrow 0 \quad \mbox{as }n\rightarrow \infty \mbox{ by Lemma 2.4}. \end{aligned}$$

 □

### Corollary 3.3

*Let* $p\geq 2$ *be positive real numbers*, *and let* $N\geq 1$ *be a positive integer*. *Suppose that* $\{X_{n}, n\geq 1\}$ *is a sequence of* $\mathbb{R}^{d}$-*valued ANA random vectors with mixing coefficients* $\rho^{-}(s)$ *such that* $\rho^{-}(N)<(\frac{1}{6p})^{p/2}$. *If* $\{X_{n}, n\geq 1\}$ *are identically distributed random vectors with* $E\Vert X_{1} \Vert ^{p}<\infty $, *then*, *for any* $\delta >\frac{1}{2}$, (3.2) *holds*.

A sequence of random vectors $\{X_{n}, n\geq 1\}$ is said to converge completely to a constant *a* if for any $\epsilon >0$,

$$ \sum_{n=1}^{\infty }P\bigl(\Vert X_{n}-a \Vert >\epsilon \bigr)< \infty . $$

In this case, we write $X_{n} \rightarrow a$ completely. This notion was given by Hsu and Robbins [11]. Note that the complete convergence implies the almost sure convergence in view of the Borel–Cantelli lemma.

The following theorem provides an extension of Theorem 4.2 of Yuan and Wu [5] for ANA random variables in $\mathbb{R}^{1}$ to random vectors in $\mathbb{R}^{d}$.

### Theorem 3.4

*Let* $p\geq 2$ *be positive real numbers*, *and let* $N\geq 1$ *be a positive integer*. *Suppose that* $\{X_{n}, n\geq 1\}$ *is a sequence of* $\mathbb{R}^{d}$-*valued ANA random vectors with mixing coefficients* $\rho^{-}(s)$ *such that* $\rho^{-}(N)<(\frac{1}{6p})^{p/2}$. *If* $\{X_{n}, n\geq 1\}$ *satisfies* (3.1), *then*, *for any* $\delta > \frac{1}{2}$,

3.4 $$ n^{-\delta }\max_{1\leq i \leq n}\Vert S_{i}-ES_{i} \Vert \rightarrow 0\quad \textit{completely}. $$

### Proof

By Lemma 2.3, Lemma 2.5, Hölder’s inequality, and the proof of Theorem 3.1 we obtain

3.5 $$\begin{aligned}& \sum_{n=1}^{\infty }E\Bigl(n^{-\delta } \max_{1\leq i\leq n}\Vert S_{i}-ES_{i} \Vert \Bigr)^{p} \\& \quad \ll \sum_{n=1}^{\infty }n^{-p\delta -1+p/2}\sum _{k=1}^{n} E\Vert X_{k} \Vert ^{p} \quad \bigl(\mbox{see (3.3)}\bigr) \\& \quad \ll \sum_{k=1}^{\infty }E\Vert X_{k} \Vert ^{p} \sum_{n=k}^{\infty }n^{-p \delta -1+p/2} \\& \quad \leq \sum_{k=1}^{\infty }k^{-p\delta -1+p/2} E \Vert X_{k} \Vert ^{p} \\& \quad \leq \sup_{n\geq 1}\frac{1}{n}\sum _{k=1}^{n} E\Vert X_{k} \Vert ^{p} \sum_{n=1}^{\infty }n^{-p\delta -1+p/2}\quad \bigl(\mbox{by Lemma~2.5}\bigr) \\& \quad < \infty \quad \Bigl(\mbox{since }{-}p\delta -1+\frac{p}{2}< -1\Bigr). \end{aligned}$$

Hence (3.4) holds. □

### Remark

Note that the proof of Theorem 3.4 is a little different from that of Theorem 4.2 in Yuan and Wu [5].

As applications of Theorem 3.4, we introduce two results that are not present in Yuan and Wu [5].

### Theorem 3.5

*Let* $p\geq 2$ *be positive real numbers*, *and let* $N\geq 1$ *be a positive integer*. *Suppose that* $\{X_{n}, n\geq 1\}$ *is a sequence of* $\mathbb{R}^{d}$-*valued ANA random vectors with mixing coefficients* $\rho^{-}(s)$ *such that* $\rho^{-}(N)<(\frac{1}{6p})^{p/2}$. *If* $\{X_{n}, n\geq 1\}$ *is weakly upper bounded by a random vector* *X* *with* $E\Vert X \Vert ^{p}<\infty $, *then*, *for any* $\delta >\frac{1}{2}$, (3.4) *holds*.

### Proof

As in the proof of Theorem 3.4, we obtain

$$\begin{aligned}& \sum_{n=1}^{\infty }E\Bigl(n^{-\delta } \max_{1\leq i\leq n}\Vert S_{i}-ES_{i} \Vert \Bigr)^{p} \\& \quad \leq \sup_{n\geq 1}\frac{1}{n}\sum _{k=1}^{n} E\Vert X_{k} \Vert ^{p} \sum_{n=1}^{\infty }n^{-p\delta -1+p/2}\quad \bigl(\mbox{see (3.5)}\bigr) \\& \quad \leq E\Vert X \Vert ^{p} \sum_{n=1}^{\infty }n^{-p\delta -1+p/2}\quad\mbox{(by Lemma~2.4)} \\& \quad < \infty \quad \Bigl(\mbox{since } {-}p\delta -1+\frac{p}{2}< -1 \mbox{ and }E\Vert X \Vert ^{p}< \infty\Bigr). \end{aligned}$$

 □

### Corollary 3.6

*Let* $p\geq 2$ *be positive real numbers*, *and let* $N\geq 1$ *be a positive integer*. *Suppose that* $\{X_{n}, n\geq 1\}$ *is a sequence of* $\mathbb{R}^{d}$-*valued ANA random vectors with mixing coefficients* $\rho^{-}(s)$ *such that* $\rho^{-}(N)<(\frac{1}{6p})^{p/2}$. *If* $\{X_{n}, n\geq 1\}$ *are identically distributed random vectors with* $E\Vert X_{1} \Vert ^{p}<\infty $, *then*, *for any* $\delta >\frac{1}{2}$, (3.4) *holds*.

## Weak law of large numbers for ANA random vectors with values in $\mathbb{R}^{d}$

In this section, we establish the weak laws of large numbers for $\mathbb{R}^{d}$-valued ANA random vectors when $p\geq 2$.

We assume that $\{X_{n}, n\geq 1\}$ is a sequence of ANA random vectors with values in $\mathbb{R}^{d}$. For $n, i \geq 1$ and $1\leq j\leq d$, we set

$$\begin{aligned}& X_{i}^{(j)}=\langle X_{i}, e_{j}\rangle , \\& Y_{ni}^{(j)}=-nI\bigl(X_{i}^{(j)}< -n \bigr)+X_{i}^{(j)}I\bigl(\bigl\vert X_{i}^{(j)} \bigr\vert \leq n\bigr)+nI\bigl(X _{i}^{(j)}>n\bigr),\quad \mbox{and} \\& Y_{ni}=\sum_{j=1}^{d} Y_{ni}^{(j)}e_{j}. \end{aligned}$$

### Theorem 4.1

*Let* $p\geq 2$ *be positive real numbers*, *and let* $N\geq 1$ *be a positive integer*. *Suppose that* $\{X_{n}, n\geq 1\}$ *is a sequence of* $\mathbb{R}^{d}$-*valued ANA random vectors with mixing coefficients* $\rho^{-}(s)$ *such that* $\rho^{-}(N)\leq r$ *and* $0\leq r<(\frac{1}{6p})^{p/2}$. *If*

4.1 $$ \lim_{n\rightarrow \infty }\sum_{i=1}^{n} \sum_{j=1}^{d} P\bigl(\bigl\vert X_{i}^{(j)} \bigr\vert >n\bigr)=0 $$

*and*

4.2 $$ \lim_{n\rightarrow \infty }\frac{\sum_{i=1}^{n}\sum_{j=1}^{d} E(\vert X_{i}^{(j)}\vert ^{p}I( \vert X_{i}^{(j)} \vert \leq n))}{n^{p}}=0, $$

*then we obtain the weak law of large numbers*

4.3 $$ \frac{1}{n}\sum_{i=1}^{n}(X_{i}-EY_{ni})\rightarrow^{p} 0\quad \textit{as }n \rightarrow \infty . $$

### Proof

By the standard method we obtain

4.4 $$\begin{aligned} P\Biggl(\frac{1}{n}\Biggl\Vert \sum_{i=1}^{n}(X_{i}-Y_{ni}) \Biggr\Vert >\epsilon \Biggr)&\leq P\Biggl( \bigcup_{i=1}^{n}(X_{i}\neq Y_{ni}) \Biggr) \\ & \leq \sum_{i=1}^{n} P(X_{i} \neq Y_{ni})=\sum_{i=1}^{n}\sum_{j=1} ^{d} P\bigl(X_{i}^{(j)} \neq Y_{ni}^{(j)}\bigr) \\ & =\sum_{i=1}^{n}\sum _{j=1}^{d} P\bigl(\bigl\vert X_{i}^{(j)} \bigr\vert >n\bigr)\rightarrow 0 \quad \mbox{as }n\rightarrow \infty \ \bigl(\mbox{by (4.1)}\bigr). \end{aligned}$$

Thus

4.5 $$ \frac{1}{n}\sum_{i=1}^{n}(X_{i}-Y_{ni}) \rightarrow^{p} 0\quad \mbox{as }n\rightarrow \infty . $$

Next, we will show that

4.6 $$ \frac{1}{n}\sum_{i=1}^{n}(Y_{ni}-EY_{ni}) \rightarrow^{p} 0\quad \mbox{as }n\rightarrow \infty . $$

It is well known that, for all $n\geq 1$, $\{Y_{ni}-EY_{ni}, i\geq 1 \}$ is a sequence of $\mathbb{R}^{d}$-valued ANA random vectors by Lemma 2.1. Then, by the Markov inequality, Hölder’s inequality, and Lemma 2.3 we have

4.7 $$\begin{aligned} &P\Biggl(\frac{1}{n}\Biggl\Vert \sum_{i=1}^{n}(Y_{ni}-EY_{ni}) \Biggr\Vert >\epsilon \Biggr) \\ &\quad \leq \frac{1}{\epsilon^{p} n^{p}}E\Biggl\Vert \sum_{i=1}^{n} (Y_{ni}-EY_{ni}) \Biggr\Vert ^{p} \\ &\quad \leq \frac{C}{n^{p}}\sum_{i=1}^{n} \sum_{j=1}^{d} E\bigl\vert Y_{ni}^{(j)} \bigr\vert ^{p} \\ &\quad \leq \frac{C}{n^{p}}\sum_{i=1}^{n}\sum_{j=1}^{d} n^{p} P\bigl(\bigl\vert X_{i}^{(j)}\bigr\vert >n\bigr)+\frac{C}{n^{p}}\sum_{i=1}^{n} \sum _{j=1}^{d} E\bigl( \bigl\vert X_{i}^{(j)}\bigr\vert ^{p}I\bigl( \bigl\vert X_{i}^{(j)} \bigr\vert \leq n\bigr)\bigr) \\ &\quad \leq C\sum_{i=1}^{n} \sum_{j=1}^{d} P\bigl(\bigl\vert X_{i}^{(j)} \bigr\vert >n\bigr)+\frac{C}{n ^{p}}\sum_{i=1}^{n} \sum_{j=1}^{d} E\bigl(\bigl\vert X_{i}^{(j)} \bigr\vert ^{p}I\bigl(\bigl\vert X_{i}^{(j)} \bigr\vert \leq n\bigr)\bigr) \\ &\quad \rightarrow 0 \quad \mbox{as }n\rightarrow \infty \ \bigl(\mbox{by (4.1) and (4.2)}\bigr), \end{aligned}$$

which yields (4.6). Combining (4.5) and (4.6), the WLLN (4.3) follows. The proof is complete. □

### Theorem 4.2

*Let* $p\geq 2$ *be positive real numbers*, *and let* $N\geq 1$ *be a positive integer*. *Suppose that* $\{X_{n}, n\geq 1\}$ *is a sequence of* $\mathbb{R}^{d}$-*valued CANA random vectors with mixing coefficients* $\rho^{-}(s)$ *such that* $\rho^{-}(N)\leq r$ *and* $0\leq r < (\frac{1}{6p})^{\frac{p}{2}}$. *If* $\{X_{n}, n\geq 1\}$ *is coordinatewise weakly upper bounded by a random vector* *X* *with*

4.8 $$ \lim_{n\rightarrow \infty }\sum_{j=1}^{d} n^{p-1}P\bigl(\bigl\vert X^{(j)} \bigr\vert >n\bigr)=0, $$

*then the WLLN* (4.3) *holds*.

### Proof

We first show that (4.5) holds. By (1.3) and (4.4) we obtain

4.9 $$\begin{aligned} &P\Biggl(\frac{1}{n}\Biggl\Vert \sum_{i=1}^{n}(X_{i}-Y_{ni}) \Biggr\Vert >\epsilon \Biggr) \\ &\quad \leq P\Biggl( \bigcup_{i=1}^{n}(X_{i} \neq Y_{ni})\Biggr) \\ &\quad \leq \sum_{i=1}^{n} \sum_{j=1}^{d} P\bigl(\bigl\vert X_{i}^{(j)} \bigr\vert >n\bigr) \\ &\quad \leq C\sum_{j=1}^{d} nP\bigl(\bigl\vert X^{(j)} \bigr\vert >n\bigr)\quad \bigl(\mbox{by (1.3)}\bigr) \\ &\quad \leq C\sum_{j=1}^{d} n^{p-1}P\bigl(\bigl\vert X^{(j)} \bigr\vert >n\bigr) \\ &\quad =o(1) \quad \mbox{by (4.8)}, \end{aligned}$$

which yields (4.5). It remains to show that (4.6) holds.

Since for all $n\geq 1$, $\{Y_{ni}-EY_{ni}, i\geq 1\}$ is a sequence of $\mathbb{R}^{d}$-valued ANA random vectors, by Lemma 2.1, Lemma 2.3, and (1.3) we have

$$\begin{aligned}& P\Biggl(\max_{1\leq k\leq n}\frac{1}{n}\Biggl\Vert \sum _{i=1}^{k} (Y_{ni}-EY_{ni}) \Biggr\Vert >\epsilon \Biggr) \\& \quad \leq n^{-p}E\Biggl(\max_{1\leq k\leq n}\Biggl\Vert \sum_{i=1}^{k} (Y_{ni}-EY_{ni}) \Biggr\Vert \Biggr)^{p}\quad (\mbox{by the Markov inequality}) \\& \quad \leq \frac{C}{n^{p}}\sum_{i=1}^{n} \sum_{j=1}^{d} E\bigl\vert Y_{ni}^{(j)} \bigr\vert ^{p}\quad \bigl(\mbox{see (4.7)}\bigr) \\& \quad \leq \frac{C}{n^{p}}\sum_{i=1}^{n} \sum_{j=1}^{d} n^{p} P\bigl(\bigl\vert X_{i}^{(j)} \bigr\vert >n\bigr)+\frac{C}{n^{p}}\sum _{i=1}^{n} \sum _{j=1}^{d} E\bigl( \bigl\vert X_{i}^{(j)} \bigr\vert ^{p}I\bigl( \bigl\vert X_{i}^{(j)} \bigr\vert \leq n\bigr)\bigr) \\& \quad = \frac{C}{n^{p-1}}\sum_{j=1}^{d} n^{p} P\bigl(\bigl\vert X^{(j)} \bigr\vert >n\bigr)+ \frac{C}{n ^{p-1}}\sum_{j=1}^{d} E\bigl(\bigl\vert X^{(j)} \bigr\vert ^{p}I\bigl(\bigl\vert X^{(j)} \bigr\vert \leq n\bigr)\bigr) \\& \quad = \frac{C}{n^{p-1}}\sum_{j=1}^{d} \int_{0}^{n} x^{p-1}P\bigl(\bigl\vert X^{(j)} \bigr\vert >x\bigr)\,dx\quad\mbox{(by integration by parts)} \\& \quad \leq \frac{C}{n^{p-1}}\sum_{j=1}^{d} \sum_{k=0}^{n-1} \int_{k}^{k+1} x^{p-1}P\bigl(\bigl\vert X^{(j)} \bigr\vert >x\bigr)\,dx \\& \quad \leq \frac{C}{n^{p-1}}\sum_{j=1}^{d} \sum_{k=1}^{n}\bigl((k+1)^{p}-k^{p} \bigr)P\bigl(\bigl\vert X^{(j)} \bigr\vert >k\bigr) \\& \quad \leq \frac{C}{n^{p-1}}\sum_{k=1}^{n} \sum_{j=1}^{d} \bigl(k^{p-1} P \bigl(\bigl\vert X^{(j)} \bigr\vert >k\bigr)\bigr) \rightarrow^{p} 0\quad \mbox{as }n\rightarrow \infty \mbox{ by (4.8)}, \end{aligned}$$

which yields (4.6). Combining (4.5) and (4.6), we obtain the WLLN (4.3). Hence the proof is complete. □

### Remark

Suppose that $\{X_{n}, n\geq 1\}$ is a sequence of $\mathbb{R}^{d}$-valued CNA random vectors. If $\{X_{n}, n\geq 1\}$ is coordinatewise weakly upper bounded by a random vector *X* with $\lim_{n\rightarrow \infty }\sum_{j=1}^{d} nP(\vert X^{(j)} \vert >n)=0$, then the WLLN (4.3) holds.

### Corollary 4.3

*Let* $N\geq 1$ *be a positive integer*, *and let* $p\geq 2$. *Suppose that* $\{X_{n}, n\geq 1\}$ *is a sequence of* $\mathbb{R}^{d}$-*valued ANA random vectors with mixing coefficient* $\rho^{-}(s)$ *such that* $\rho^{-}(N)\leq r$ *and* $0\leq r<( \frac{1}{6p})^{\frac{p}{2}}$. *If* $\{X_{n}, n\geq 1\}$ *is a sequence of identically distributed random vectors with*

4.8′ $$ \lim_{n\rightarrow \infty }\sum_{j=1}^{d} n^{p-1}P\bigl(\bigl\vert X_{1}^{(j)} \bigr\vert >n\bigr)=0, $$

*then* (4.3) *holds*.

### Theorem 4.4

*Let* $p\geq 2$, *and let* $N\geq 1$ *be an integer*. *Suppose that* $\{X_{n}, n\geq 1\}$ *is a sequence of mean zero* $\mathbb{R}^{d}$-*valued ANA random vectors with mixing coefficients* $\rho^{-}(s)$ *such that* $\rho^{-}(N)< r$ *and* $0\leq r < (\frac{1}{6p})^{ \frac{p}{2}}$. *If* $\{X_{n}, n\geq 1\}$ *is coordinatewise weakly upper bounded by a random vector* *X* *with*

4.10 $$ \sum_{j=1}^{d} E\bigl(\bigl\vert X^{(j)} \bigr\vert ^{p-1}\bigr)< \infty , $$

*then*

4.11 $$ \frac{1}{n}\sum_{i=1}^{n} X_{i} \rightarrow^{p} 0 \quad \textit{as }n\rightarrow \infty. $$

### Proof

It follows from (4.10) that

4.12 $$ \lim_{n\rightarrow \infty } \sum_{j=1}^{d} E\bigl(\bigl\vert X^{(j)} \bigr\vert ^{p-1}I\bigl(\bigl\vert X^{(j)} \bigr\vert >n\bigr)\bigr)=0, $$

which yields

4.13 $$\begin{aligned} &\Biggl\Vert \frac{1}{n}\sum_{i=1}^{n} EY_{ni} \Biggr\Vert \\ &\quad \leq \frac{1}{n}\sum _{i=1}^{n} \Vert EY_{ni} \Vert \\ &\quad \leq \frac{1}{n}\sum_{j=1}^{d} \sum_{i=1}^{n} \bigl\vert EY_{ni}^{(j)}\bigr\vert \\ &\quad \leq \frac{1}{n}\sum_{j=1}^{d} \sum_{i=1}^{n} \bigl\vert E\bigl(X_{i}^{(j)}I\bigl( \bigl\vert X _{i}^{(j)}\bigr\vert \leq n\bigr)\bigr) \bigr\vert +\frac{1}{n}\sum _{j=1}^{d} \sum_{i=1}^{n} nP\bigl(\bigl\vert X_{i}^{(j)} \bigr\vert > n\bigr) \\ &\quad =\frac{1}{n}\sum_{j=1}^{d} \sum_{i=1}^{n} \bigl\vert E\bigl(X_{i}^{(j)}I\bigl( \bigr\vert X_{i}^{(j)}\bigr\vert >n\bigr)\bigr) \bigr\vert \\ &\quad \quad {}+\frac{1}{n}\sum_{j=1}^{d} \sum_{i=1}^{n} nP\bigl(\bigl\vert X_{i}^{(j)} \bigr\vert > n\bigr)\quad (\mbox{by $EX_{i}=0$}) \\ &\quad \leq \sum_{j=1}^{d} E\bigl(\bigl\vert X^{(j)} \bigr\vert I\bigl(\bigl\vert X^{(j)} \bigr\vert >n \bigr)\bigr) +\sum_{j=1}^{d} nP\bigl( \bigl\vert X^{(j)} \bigr\vert > n\bigr)\quad\bigl(\mbox{by (1.3)}\bigr) \\ &\quad \leq C\sum_{j=1}^{d} E\bigl(\bigl\vert X^{(j)} \bigr\vert ^{p-1}I\bigl(\bigl\vert X^{(j)} \bigr\vert >n\bigr)\bigr) +C\sum_{j=1}^{d} n^{p-1}P\bigl(\bigl\vert X^{(j)} \bigr\vert > n\bigr) \\ &\quad \leq 2C\sum_{j=1}^{d} E\bigl(\bigl\vert X^{(j)} \bigr\vert ^{p-1}I\bigl(\bigl\vert X^{(j)}\bigr\vert >n\bigr)\bigr)\rightarrow ^{p} 0 \quad \mbox{as }n\rightarrow \infty \ \bigl(\mbox{by (4.12)}\bigr). \end{aligned}$$

It remains to prove (4.3).

Since (4.12) implies (4.8), (4.3) follows from Theorem 4.2. Thus the proof is complete. □

### Remark

Suppose that $\{X_{n}, n\geq 1\}$ is a zero-mean sequence of $\mathbb{R}^{d}$-valued NA random vectors. If $\{X_{n}, n \geq 1\}$ is coordinatewise weakly upper bounded by a random vector *X* with $\sum_{j=1}^{d} E\vert X^{(j)} \vert <\infty $, then (4.11) holds.

### Corollary 4.5

*Let* $N\geq 1$ *be an integer*, *and let* $p\geq 2$. *Suppose that* $\{X_{n}, n\geq 1\}$ *is a sequence of* $\mathbb{R}^{d}$-*valued ANA random vectors with mixing coefficient* $\rho^{-}(s)$ *such that* $\rho^{-}(N)\leq r$ *and* $0\leq r<( \frac{1}{6p})^{\frac{p}{2}}$. *If* $\{X_{n}, n\geq 1\}$ *is a sequence of identically distributed random vectors with* $EX_{1}=0$ *and*

4.10′ $$ \sum_{j=1}^{d} E\bigl(\bigl\vert X_{1}^{(j)} \bigr\vert ^{p-1}\bigr)< \infty , $$

*then we obtain the WLLN* (4.11).

### Proof

The proof follows by substituting $X^{(j)}$ by $X_{1}^{(j)}$ in the proof of Theorem 4.4. □

## Conclusions

We generalized the $L_{p}$-convergence and complete convergence results of Yuan and Wu [5] from $\mathbb{R}^{1}$-valued ANA random variables to $\mathbb{R}^{d}$-valued random vectors by using a Rosenthal-type inequality. We also established weak laws of large numbers for CANA random vectors under $p\geq 2$. As applications, we obtained some $L_{p}$-convergence and complete convergence results that are not present in Yuan and Wu [5] even when $d=1$.
